# Targeting TaNSUN2 provides a potential route to combine antiviral resistance with improved productivity in wheat

**DOI:** 10.3389/fmicb.2026.1874667

**Published:** 2026-08-31

**Authors:** Qingxiao Jia, Yonglu Zhou, Wenjun Yu, Junjie Li

**Affiliations:** State Key Laboratory of Agriculture and Forestry Biosecurity, Center for Genetic Improvement, College of Plant Protection, Fujian Agriculture and Forestry University, Fuzhou, China

**Keywords:** antiviral, Chinese wheat mosaic virus, pathobiome, RNA 5-methylcytosine (m5C), TaNSUN2

## Introduction

Research on crop disease has shifted from a single pathogen-host framework toward a multifactorial host-microbiota-environment framework. This transition, captured by the pathobiome concept, redirects attention from individual pathogens to the broader biological networks that condition disease establishment, progression, and outcome (Bass et al., 2019). In plants, disease can therefore be considered within a microbiota-aware framework in which pathogen success is shaped by host genotype, environment, and surrounding microbial consortia, including their ecological reassembly during infection.

**Figure 1:**
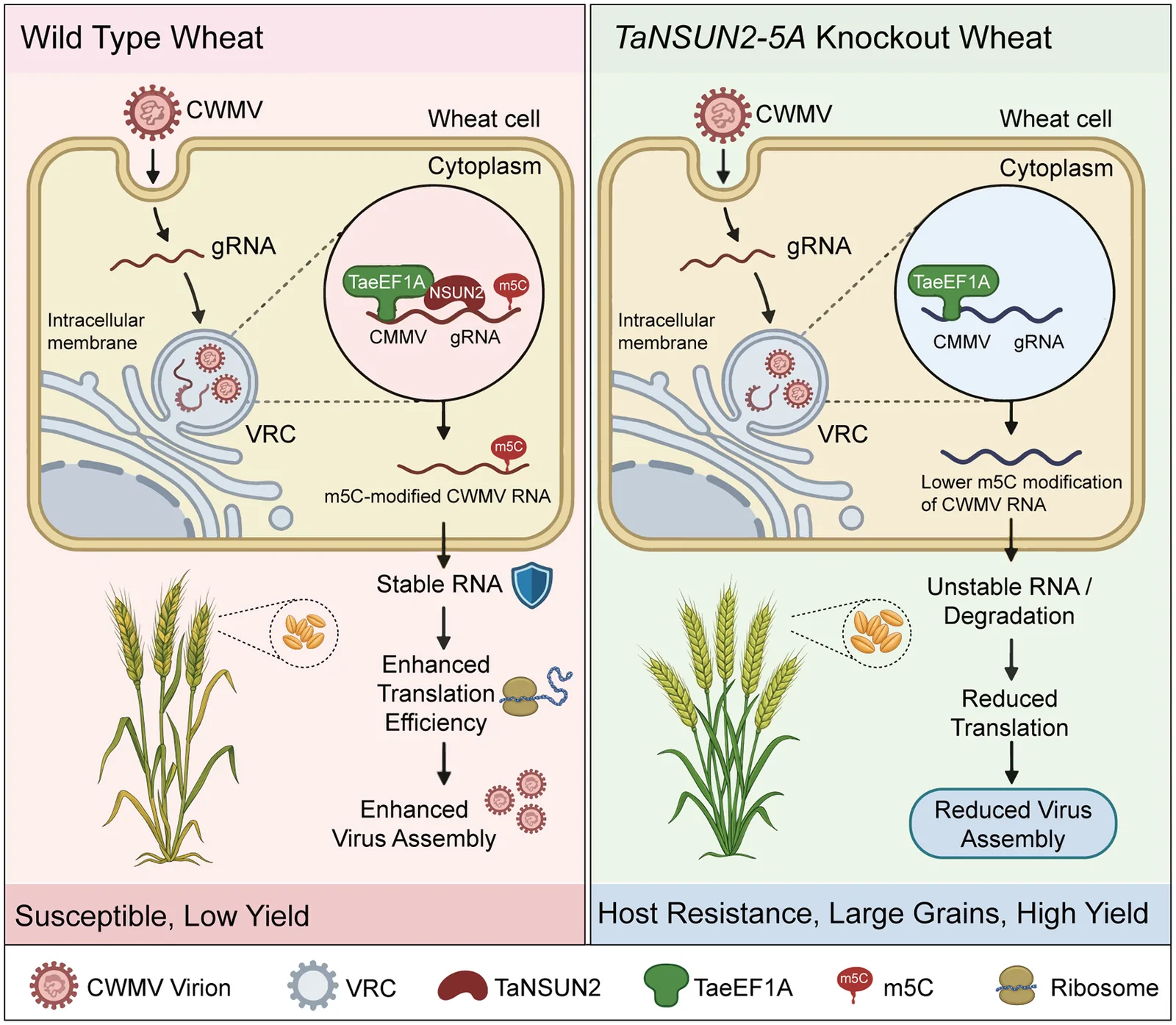
Working model of TaNSUN2-mediated CWMV susceptibility and TaNSUN2-5A-associated yield-related traits in wheat. A working model summarizing the findings of Jiang et al. (2026). During CWMV infection, the host elongation factor TaeEF1A recruits TaNSUN2 to viral replication complexes (VRCs). TaNSUN2 catalyzes m5C modification of viral RNAs, thereby increasing RNA stability and translational efficiency and promoting viral replication and assembly. By contrast, *TaNSUN2-5A* knockout reduces viral RNA m5C modification, destabilizes CWMV RNAs, impairs translation and viral accumulation, and confers resistance together with larger grains and higher yield per plant under the reported conditions.

Within this broader context, host susceptibility factors are important because they can influence pathogen fitness as well as the infection-prone physiological state of the host. Jiang et al. (2026) identify the wheat RNA 5-methylcytosine (m5C) methyltransferase TaNSUN2 as a host factor co-opted by Chinese wheat mosaic virus (CWMV), and show that weakening this dependency enhances antiviral resistance while improving yield-related traits (Figure 1) (Jiang et al., 2026). The study motivates two linked questions: how does CWMV redirect TaNSUN2 to control viral RNA fate, and can homoeolog-specific perturbation reduce this dependency without imposing a productivity penalty? The working hypothesis developed here is that a discrete TaeEF1A-TaNSUN2 recruitment module, rather than the entirety of TaNSUN2 function, constitutes a tractable susceptibility node. From a pathobiome-aware perspective, this is a virus-exploited epitranscriptomic host-dependency module; however, the study does not directly demonstrate restructuring of the wider pathobiome.

CWMV is a soil-borne, bipartite positive-sense RNA furovirus that infects wheat and is transmitted to roots by the obligate plasmodiophorid *Polymyxa graminis* (Diao et al., 1999). Infection produces chlorotic mosaic symptoms and stunting in winter wheat, while long-lived vector resting spores make eradication from infested soil difficult. Resistant cultivars therefore remain the most practical management option, which gives particular agricultural relevance to host susceptibility factors that can be modified without compromising yield.

## m5C machinery enters the plant-virus arena

RNA modifications are central post-transcriptional regulators of RNA metabolism and gene expression in eukaryotes (Wei and He, 2026). Among them, m5C influences RNA stability, export, translation, and stress adaptation (Nachtergaele and He, 2018; Zhang et al., 2020). Chemically, m5C is installed by S-adenosyl-L-methionine-dependent RNA cytosine-5 methyltransferases through a conserved catalytic cysteine and a covalent enzyme-RNA intermediate (Nachtergaele and He, 2018; Zhang et al., 2020). In mammals, NSUN2 is a major mRNA m5C writer, whereas ALYREF and YBX1 recognize or bind m5C-modified transcripts to regulate export and stability (Yang et al., 2017; Chen et al., 2019). In plants, transcriptome-wide analyses have detected m5C in coding and noncoding RNAs and linked it to development and environmental responses. In Arabidopsis, TRM4B functions as an RNA m5C writer; *trm4b* mutants show reduced m5C, altered stability of target transcripts, and root-development defects (Cui et al., 2017). In rice, OsNSUN2 maintains mRNA m5C methylation and supports growth under elevated temperature (Tang et al., 2020). Plant m5C readers and erasers remain less completely defined, and it was unclear whether a virus could redirect a host m5C writer to viral RNA and whether such exploitation could be disrupted without an agronomic penalty.

Plant-virus epitranscriptomics has so far been shaped largely by studies of N6-methyladenosine (m6A), which can have opposing effects in different virus-host combinations. In *Arabidopsis*, the m6A demethylase ALKBH9B promotes alfalfa mosaic virus infection and vascular movement (Martinez-Perez et al., 2017, 2021), whereas YTH-domain ECT proteins recognize m6A-marked viral RNA and contribute to antiviral restriction (Martinez-Perez et al., 2023). In wheat, TaHAKAI has context-dependent functions during wheat yellow mosaic virus infection: its m6A-writer activity can favor viral RNA accumulation, while its E3-ligase activity promotes degradation of a viral silencing suppressor and is linked to favorable spike traits (Guo et al., 2025). Recent tomato profiling further showed that tomato spotted wilt virus reshapes the host m5C landscape and that SlTRM4B stabilizes defense-related host transcripts (Zhao et al., 2025). These comparisons underscore that the outcome of an RNA modification depends on the modified substrate, the reader or effector recruited, and the viral context. The TaNSUN2 study is distinctive because it directly links m5C deposition on viral RNA with homoeolog-specific functional differentiation and favorable agronomic consequences.

Jiang et al. address this unresolved m5C question directly. CWMV does not appear to recruit TaNSUN2 on its own; instead, it co-opts the host translation elongation factor TaeEF1A, which recruits TaNSUN2 to viral replication complexes (VRCs). Within VRCs, TaNSUN2 deposits m5C on viral RNAs, increasing RNA stability and translational efficiency. m5C deposition in the viral 3′ untranslated region has two complementary effects: it strengthens the association of TaeEF1A with viral RNA to support replication and enhances coat-protein binding to promote virion assembly (Figure 1). Thus, CWMV repurposes host m5C machinery at multiple stages of infection, providing a direct example of viral exploitation of an epitranscriptomic regulator.

## Natural allelic variation weakens a critical host-virus interface

A particularly important aspect of the study is that resistance arises by weakening a defined host-virus molecular interface rather than by eliminating all TaNSUN2 activity. Natural allelic variation in TaNSUN2 reduces its affinity for TaeEF1A, thereby limiting recruitment to viral replication complexes and attenuating proviral activity without broadly disrupting host RNA metabolism. This distinction matters because resistance may be achieved not only by knocking out susceptibility genes, but also by selectively disrupting the pathogen-dependent interaction while preserving endogenous functions.

This principle aligns with a broader trend in plant disease biology: host dependency often resides in discrete molecular interfaces rather than in the entirety of a host protein's physiological role. The susceptibility-gene framework likewise emphasizes that loss or modification of host compatibility factors can provide resistance, while pleiotropic effects must be evaluated case by case (van Schie and Takken, 2014; Pavan et al., 2010). TaNSUN2 is therefore especially relevant to breeding because naturally occurring or engineered variants could preserve core endogenous functions while diminishing pathogen exploitation. A related principle is illustrated by the Rice grassy stunt virus (RGSV) P3-D14/SL pathway, in which infection depends on a specific interaction with a host hormone receptor to hijack strigolactone signaling and suppress antiviral RNA interference (Yang et al., 2026). Precise modification of this interaction interface can confer robust resistance without substantial growth or yield penalties.

## Combining enhanced antiviral defense with favorable yield-related traits

Particularly noteworthy is the agronomic outcome of perturbing TaNSUN2. Jiang et al. report that knockout of *TaNSUN2-5A* markedly enhances CWMV resistance, increases thousand-grain weight by approximately 5.9 g, improves grain length and width, and raises yield per plant by more than 14%. Under the genetic background and conditions examined, enhanced antiviral resistance therefore did not require a detectable productivity trade-off. This result supports a cautious interpretation: *TaNSUN2-5A* is a promising route for combining resistance with favorable yield-related traits, although it does not yet establish a general mechanistic uncoupling of susceptibility from productivity.

One plausible explanation for this favorable outcome is homoeolog specialization. Because the three TaNSUN2 copies are not functionally equivalent, selective perturbation of *TaNSUN2-5A* may strengthen antiviral defense while the other copies retain essential endogenous functions. Polyploid redundancy therefore becomes an opportunity for allele-specific or copy-specific intervention rather than only an obstacle to genotype-phenotype analysis. Such subfunctionalization could buffer developmental costs and permit finer adjustment than would be possible in a single-copy system. Nevertheless, the causal contribution of homoeolog specialization to the favorable yield phenotype remains to be resolved.

Viewed in this way, the study does more than identify a susceptibility factor. It offers a potential route toward precision breeding in which resistance is combined with preserved or improved yield. This favorable combination makes TaNSUN2 relevant beyond the immediate wheat-CWMV system. Nevertheless, broader applicability will require direct testing across additional viruses, wheat backgrounds, and environmental conditions.

## TaNSUN2 as a candidate host-dependency node in a pathobiome-aware framework

Within a pathobiome-aware framework, host regulators that alter both pathogen fitness and host physiology can be considered candidate nodes of disease compatibility, although such network-level effects have not been demonstrated for TaNSUN2. This framing is relevant in wheat because the three *TaNSUN2* homoeologs are not equivalent: *TaNSUN2-5A* is preferentially associated with the viral response, whereas *TaNSUN2-5B* appears more closely linked to root development. TaNSUN2 may therefore be viewed as a dosage-sensitive regulatory node at the intersection of epitranscriptomic control, polyploid genome organization, and disease compatibility. Targeting one homoeolog could potentially shift viral compatibility while other copies maintain the broader developmental program. This remains a hypothesis-generating interpretation and should not be taken as evidence that TaNSUN2 remodels rhizosphere or phyllosphere communities.

Parallel evidence from tropical polyploid and clonally propagated crops supports the broader breeding logic. Cultivated banana germplasm arose through hybridization and polyploidization and is characterized by high heterozygosity, clonal propagation, and reduced fertility, all of which complicate conventional resistance breeding (Berankova et al., 2024). Nevertheless, susceptibility-focused genome editing has proved effective: editing the banana *DMR6* ortholog enhanced resistance to bacterial disease (Tripathi et al., 2021), and targeted knockout of *MusaENODL3* increased resistance to *Xanthomonas* wilt (Ntui et al., 2024). These studies do not identify TaNSUN2-like epitranscriptomic regulators, but they show that host-encoded vulnerability nodes can be reconfigured in genetically complex crops. The comparison therefore concerns a shared breeding logic, not a shared TaNSUN2 pathway.

A related pattern is emerging in sugarcane, another highly polyploid crop in which conventional breeding is constrained by genome complexity and redundancy (Altpeter, 2025). Recent studies identify actionable host-side targets, including the negative regulators ScWRKY2 and ScCAX4 (Wang et al., 2025; Zhang et al., 2024), and virus-exploited host factors such as ScHSP17.5 and ScHSP17.9A, which facilitate sugarcane mosaic virus replication (Yuan et al., 2026). Together, these findings suggest that crop improvement in complex genomes may benefit from identifying and weakening recurrent host dependencies. In this context, TaNSUN2 should be viewed as a candidate host-dependency node whose possible pathobiome-level relevance remains a testable hypothesis.

## Discussion

This work provides one of the first functionally validated examples of an mRNA m5C methyltransferase acting as a susceptibility factor in a plant viral pathosystem. It also highlights host epitranscriptomic regulators as a source of agronomically useful variation and raises the possibility that TaNSUN2 promotes infection through both direct and indirect routes. In addition to methylating viral RNA, TaNSUN2 may modify host transcripts or cellular states that favor infection. These possibilities make TaNSUN2 a useful system for linking RNA modification, pathogen compatibility, and quantitative crop performance, but the host-transcript component remains to be tested.

The open questions can be organized into a three-stage translational roadmap. First, mechanistic resolution should define how viral m5C sites are selected, identify the relevant readers or RNA-binding effectors, and separate catalytic effects from TaeEF1A-mediated recruitment. Second, biological validation should test *TaNSUN2-5A* alleles across CWMV isolates, additional wheat backgrounds, and field environments, while measuring pleiotropic effects on development, yield, and the host transcriptome. Third, breeding translation should mine natural allelic diversity, edit discrete interaction surfaces or catalytic features, and evaluate resistance durability, viral adaptation, and performance across other viruses and polyploid crops. This sequence moves from molecular causality to genotype-by-environment validation and, only then, to deployment.

Viewed comparatively, the banana and sugarcane examples support the general feasibility of susceptibility-centered intervention in complex crop genomes, but they do not establish transferability of the TaNSUN2 mechanism. For wheat, a practical route would involve mining natural TaNSUN2 alleles, developing markers for favorable homoeolog-specific variants, and testing targeted editing of interaction interfaces or individual homoeologs. Each strategy should be evaluated for pleiotropy, resistance durability, and potential viral adaptation (Sanfacon, 2015).

By identifying TaNSUN2 as a virus-exploited host factor with breeding relevance, Jiang et al. provide a concrete example in which weakening a pathogen-dependent module enhances resistance and improves yield-related traits in wheat. The study positions epitranscriptomic regulation as a promising, but still experimentally bounded, target for precision breeding. Whether the same principle extends beyond the wheat-CWMV system now requires comparative and field-scale validation.
